# Anti-N-methyl-D-aspartate receptor(NMDAR) antibody encephalitis presents in atypical types and coexists with neuromyelitis optica spectrum disorder or neurosyphilis

**DOI:** 10.1186/s12883-016-0787-9

**Published:** 2017-01-05

**Authors:** Kaiyu Qin, Wenqing Wu, Yuming Huang, Dongmei Xu, Lei Zhang, Bowen Zheng, Meijuan Jiang, Cheng Kou, Junhua Gao, Wurong Li, Jinglin Zhang, Sumei Wang, Yanfei Luan, Chaoling Yan, Dan Xu, Xinmei Zheng

**Affiliations:** Department of neurology, Beijing Ditan Hospital, Capital Medical University, No.8 East Jing Shun Rd, Chaoyang District, Beijing, China

**Keywords:** Anti-N-methyl-D-aspartate receptor(NMDAR), Atypical types, Neuromyelitis optica spectrum disorder, Neurosyphilis

## Abstract

**Background:**

Anti-N-methyl-D-aspartate receptor (NMDAR) encephalitis is a clinically heterogeneous disorder characterized by epileptic seizures, psychosis, dyskinesia, consciousness impairments, and autonomic instability. Symptoms are always various. Sometimes it presents in milder or incomplete forms. We report 4 cases of anti-NMDAR encephalitis with incomplete forms, 3 cases of which were accompanied by neuromyelitis optica spectrum disorder or neurosyphilis respectively.

**Case presentation:**

A 33-year-old man presented with dysarthria, movement disorder and occasional seizures. He had 6 relapses in 28 years. When suffered from upper respiratory tract syndrome, he developed behavioral and consciousness impairment. Cranial MRI was normal. Viral PCR studies and oncologic work-up were negative. Anti-NMDAR antibody was detected in CSF and serum.

A 21-year-old female manifested dizziness and diplopia ten months and six months before, respectively. Both responded to steroid therapy and improved completely. This time she presented with progressive left limb and facial anesthesia, walking and holding unsteadily. Spinal cord MRI follow-up showed abnormality of medulla oblongata and cervical cord(C1). Anti-AQP4 and anti-NMDAR were positive in CSF. Steroid-pulse therapy ameliorated her symptoms.

A 37-year-old male experienced worsening vision. He was confirmed neurosyphilis since the CSF tests for syphilis were positive. Protein was elevated and the oligoclonal IgG bands(OB) and anti-NMDAR was positive in CSF. Anti-aquaporin 4(AQP4) antibodies and NMO-IgG were negative. Cranial MRI showed high FLAIR signal on frontal lobe and low T2 signal adjacent to the right cornu posterious ventriculi lateralis. Treatment for neurosyphlis was commenced with gradual improvement.

A 39-year-old male, developed serious behavioral and psychiatric symptoms. Examination showed abnormal pupils and unsteady gait. He was confirmed neurosyphilis according to the CSF tests for syphilis. Anti-NMDAR was positive in CSF and serum. Cranial MRI showed lateral ventricles and the third ventricle enlargement and signal abnormality involving bilateral temporal lobe, corona radiate and centrum semiovale. PenicillinG, pulsed methylprednisolone and intravenous immunoglobulin was administered. He was stable.

**Conclusion:**

Anti-NMDAR encephalitis can present in atypical types. When relapsing, it may present with partial aspects or with isolated symptoms of the full-blown syndrome. Anti-NMDAR encephalitis may be related to neuromyelitis optica spectrum disorder or neurosyphilis.

## Background

Anti-NMDAR encephalitis is a severe but treatable auto-immune disorder. It can present with psychosis, memory deficits, seizures, dyskinesia, involuntary movements, decreased level of consciousness, and autonomic instability. Anti-NMDAR encephalitis usually evolves through several stages: flu-like prodromal syndromes, a psychotic stage, unresponsiveness with hypoventilation, autonomic instability and dyskinesia, and eventually death or recovery [1, 2]. The clinical phases of anti-NMDA receptor encephalitis may vary in sequence, presentation, and severity [3]. Milder or incomplete forms of the disorder can occur [4]. Here we report 4 cases presenting in atypical types.

## Case presentation

Patient 1 was a 33-year-old male who was brought to our ward complaining of memory deficit, vision field with grids and stars background, and intermittent psychomotor agitation, following upper respiratory tract syndromes. His previous medical history indicated normal growth and development, except for epilepsy. He was diagnosed epilepsy during 9 years old because of paroxysmal formication responsive to antiepileptic drugs. There was no seizure until he manifested paroxysmal tongue rigidity and dysarthria for several days without any precipitating factor 16 years later. Thereafter, he developed episodes of “blank staring”. Half a month later, he had paroxysmal head and eyes turning right, trismus, and rigidity of the left limbs. During having upper respiratory tract syndromes, that was accompanied by agitation, akathisia, hyperkinetic movements, disorientation to person and place, unresponsiveness, and consciousness impairment. Though antiepileptic drugs were prescribed, there were seizures every 3–5 years.

His reflexes were normal. There were no meningeal signs and no extensor plantar response. The CSF showed a lymphocyte pleocytosis, normal protein and glucose. Viral PCR studies for herpes simplex, cytomegalovirus, rubella, and toxoplasmosis were negative. Paraneoplastic tests such as Hu, Ri, Yo, CV_2_, amphiphysin were negative, except for anti-NMDAR antibody positive in CSF and weakly positive in serum. Cranial MRI was normal.

He responded to steroid (dexamethasone 20 mg for 3 days and slow weaning of oral prednisolone) and anti-epileptic therapy. Relapse symptoms decreased after steroid received. Now he has weaned from anti-epileptic drugs for about 1 year, and there was no seizure. His blood test for anti-NMDAR-Ab became negative and CSF weakly positive when he recovered completely 1 year later. Despite an extensive oncologic work-up such as chest X-ray, pelvic and abdomen ultrasonic examination, there was no evidence of tumor, thus confirming the diagnosis of anti-NMDAR encephalitis.

Recently, he presented with paroxysmal choreiform movements of left hand, and slurring of speech (with delay of 3 years for the last relapse). His blood test for anti-NMDAR-Ab was reactive in 1:10 dilutions and CSF 1:100 dilutions. The movement disorder and dysarthria resolved after he was commenced on pulsed methylpredinisolone(500 mg). To prevent relapse, he received mycophenolate mofetil.

Patient 2, a 21-year-old female, suffered from progressive left limbs and facial anesthesia for a month, walking and holding unsteadily for 10 days. She also suffered from paroxysmal pain of facial and neck on the left side, and episodes of intractable vomiting, accompanied by anxiety. Ten months and six months before admission, she described dizziness, jittering vision, and diplopia on left gaze respectively, which responded to steroid therapy and improved completely. At her admission, examination noted fever(T:38.5 °C),facial dysesthesia on the left side,walking difficulties related to moderate superficial and deep sensory dysfunction and hemiparesis of the left limbs, difficulty in executing finger-to-nose and heel-to-knee maneuvers because of her ataxia. The left Babinski’s sign, left Hoffmann’s sign and Lhermitte’s sign were positive.

**Fig. 1:**
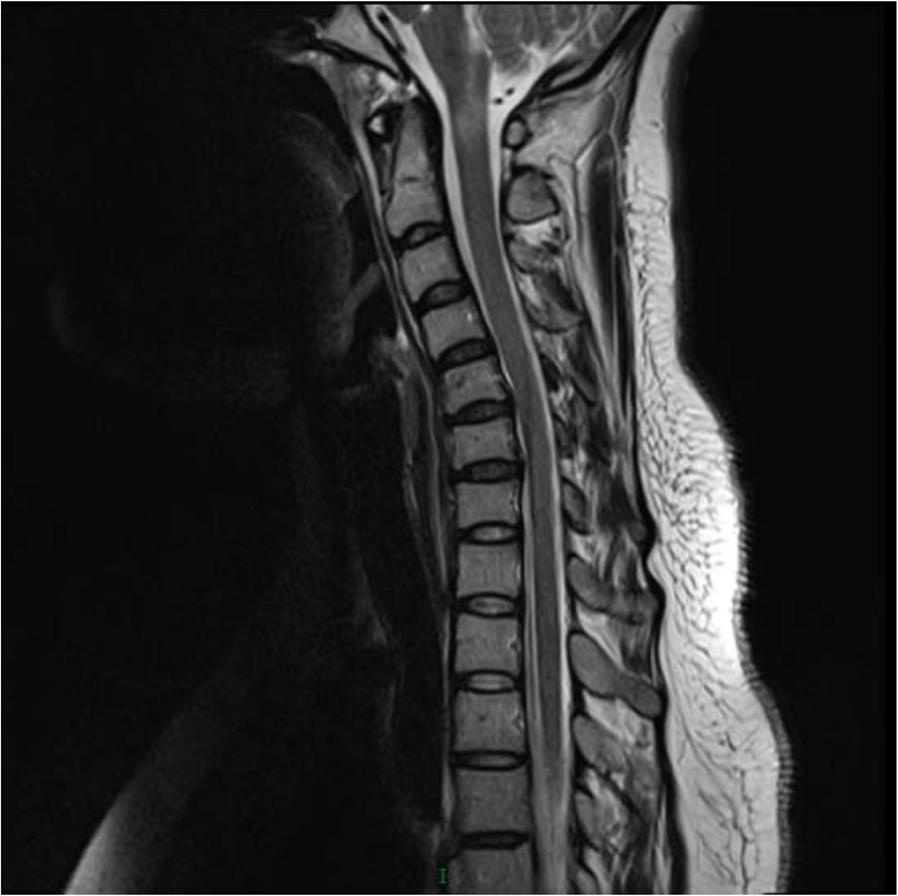
T2-weighted MRI image showing high-signal intensity from the dorsal medulla to cervical cord(C1)

Spinal cord MRI follow-up showed T2 hyperintensities and T1 hypointensities from the dorsal medulla to cervical cord(C1) (Fig. 1). Visual evoked potentials and EEG are normal. Analysis of the CSF showed almost acellular(1600/ml), normal protein and glucose concentration. OB(oligoclonal band) and MBP(myelin basic protein) are negative. Tests for paraneoplasia such as Hu, Ri, Yo, CV_2_, amphiphysin were all negative. Anti-NMDAR-Ab was positive, and anti-aquaporin 4(AQP4) antibodies weakly positive in CSF, while anti-NMDAR-Ab negative and AQP4 antibodies weakly positive in serum. Oncologic tests, including chest X-ray, pelvic and abdomen ultrasonic examination, CA125, CEA(carcinoembryonic antigen), were all negative. Autoimmune tests such as ANA(antinuclear antibodies), ENA, and thyroid function were normal. She received pulsed methylpredinisolone(500 mg). Four days after the steroid therapy, unsteadiness of the left limbs was greatly improved. Considering anti-AQP4 and anti-NMDAR were positive in CSF, we gave her azathioprine to prevent relapse. No recrudescence developed throughout three months, and MRI showed improvement in the previously observed high T2 signal abnormalities.

**Fig. 2:**
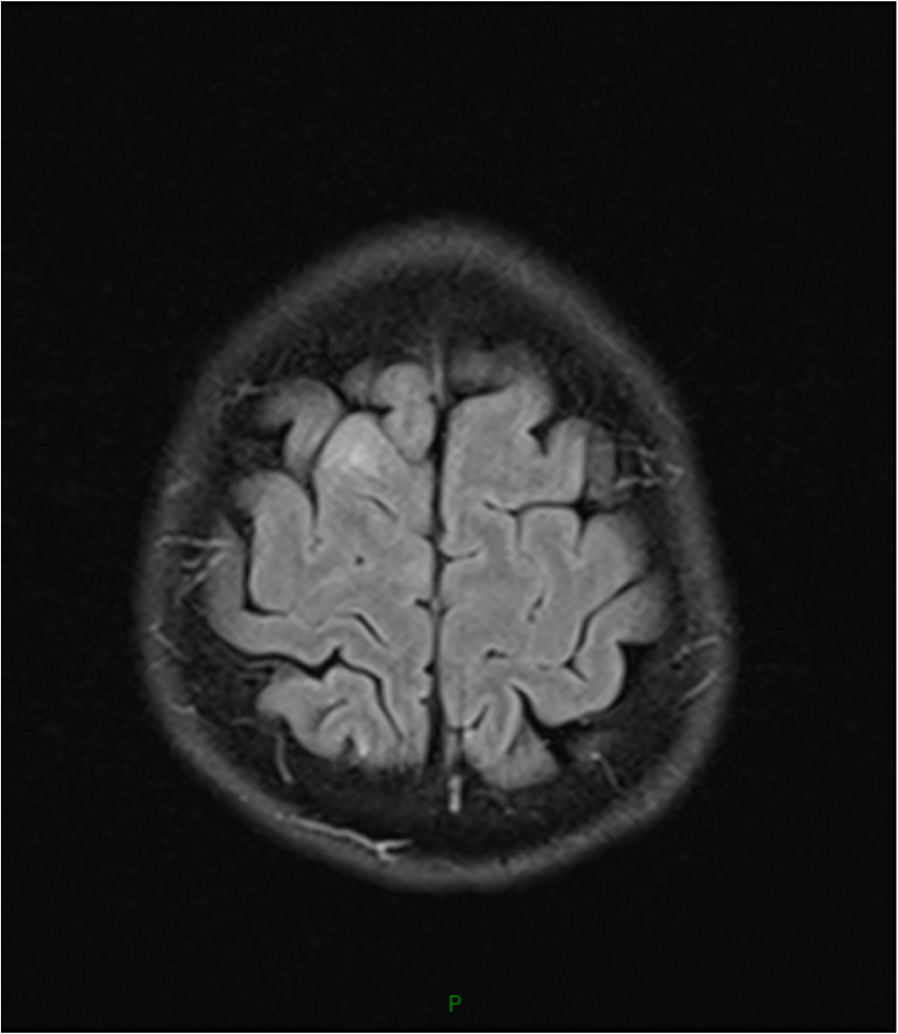
FLAIR image showing high signal on right frontal lobe

**Fig. 3:**
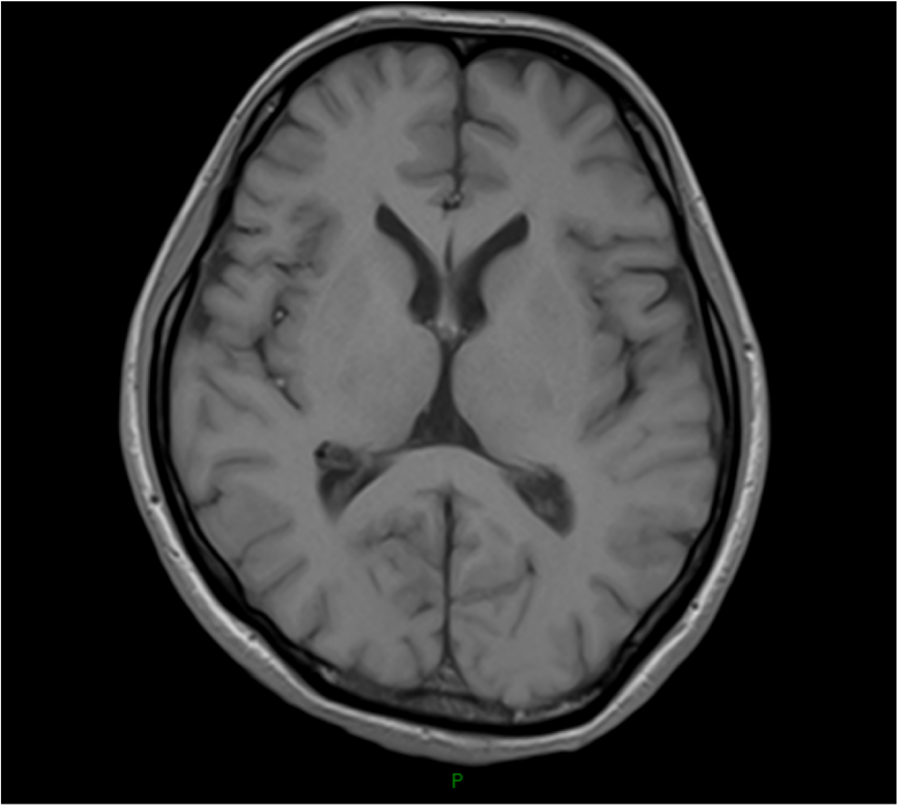
T1-weighted Cranial MRI showing low-signal adjacent to the right cornu posterious ventriculi lateralis

Patient 3, a 37-year-old male, who was diagnosed syphilis 2 years before, presented with progressively reduced vision of both eyes to 0.02 for six months. Human immunodeficiency virus(HIV) testing was negative. The CSF was almost acellular(6/mm^3^) with elevated protein 1.237 g/L, normal glucose, and tests for syphilis were positive for non-treponeal(Syphilis Toluidine Red Untreated Serum Test, TRUST reactive in 1:2 dilutions) and reactive in specific Treponema Pallidum particle agglutination and Treponema enzyme-linked immunosobent assays(IgG positive, IgM negative). The oligoclonal IgG bands(OB) and anti-NMDAR was positive in CSF. Anti-aquaporin 4(AQP4) antibodies and NMO-IgG were negative. Visual evoked potentials showed no P100 wave on the left side and delayed P100 latencies on the right side. Cranial MRI showed a focal slightly high FLAIR signal on frontal lobe (Fig. 2) and low T2 signal adjacent to the right cornu posterious ventriculi lateralis (Fig. 3). The patient was treated with intravenous penicillin G, followed by intramuscular injection of 2.4 million U of benzathine penicillin G weekly for 3 weeks. The vision was improved to 0.04 about 1 year later, and CSF tests for syphilis were negative for non-treponeal(TRUST).

**Fig. 4:**
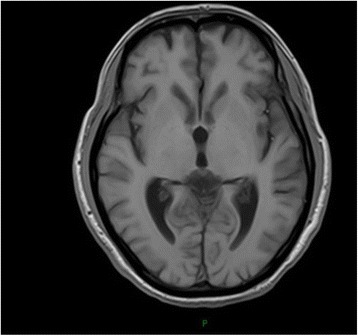
T1-weighted head MRI showing lateral ventricles and the third ventricle enlargement

**Fig. 5:**
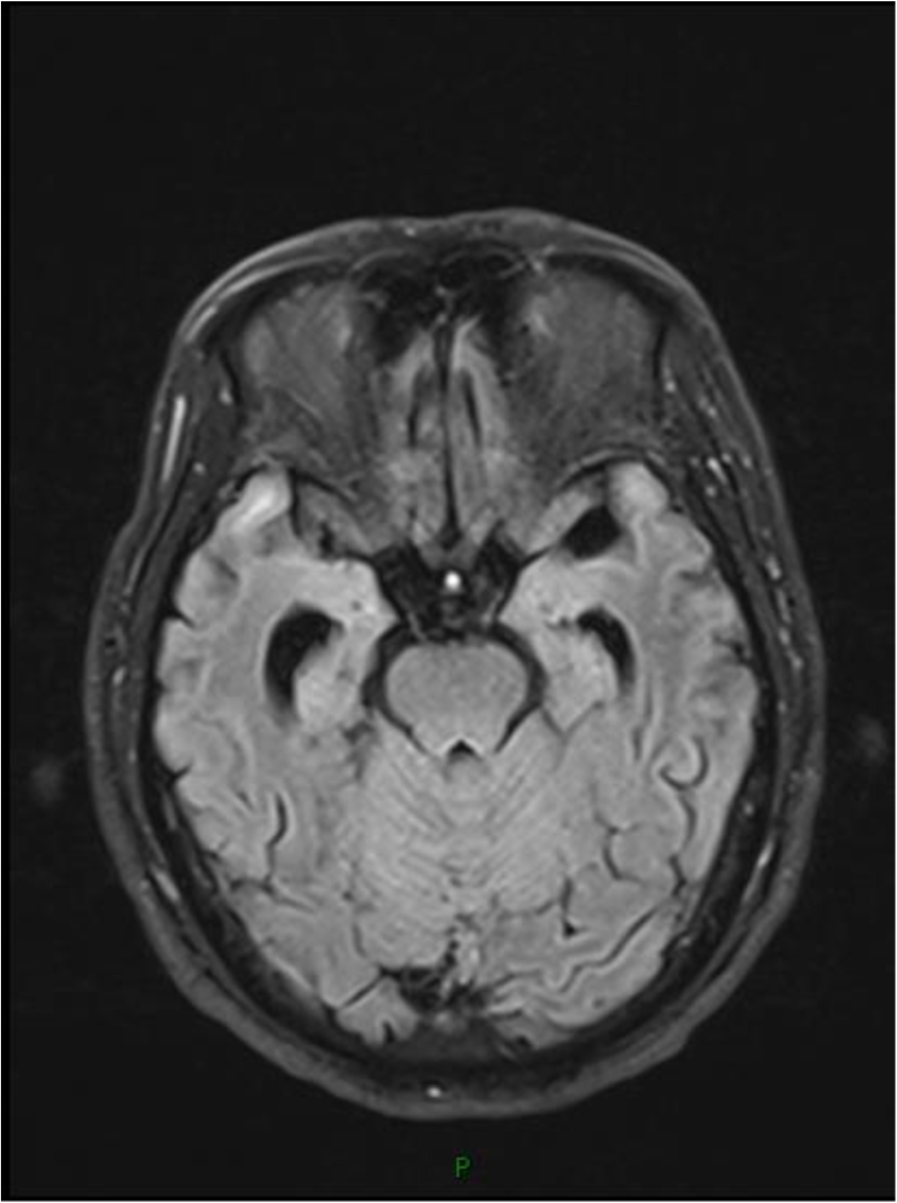
FLAIR image showing high signal on bilateral temporal lobe

**Fig. 6:**
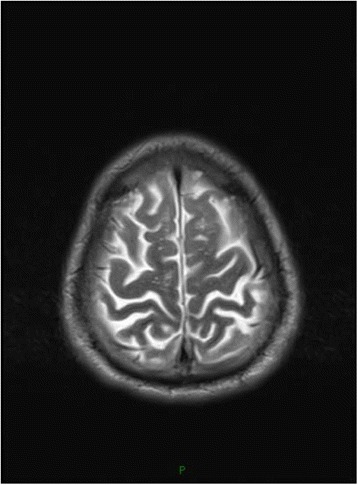
T2-weighted head MRI showing high signal of bilateral corona radiate and centrum semiovale

Patient 4, a 39-year-old male, manifested progressive attention and memory impairments and episodes of anxiety and irritability reported by family members. Examination showed under illumination, his right and left pupils were 3 and 5 mm in diameter, respectively. Both pupils were insensitive to light. His gait was unsteady. He swayed a little from side to side when he stood erect with his eyes closed. HIV testing was negative. Blood tests for syphilis were positive for non-treponeal(TRUST reactive in 1:32 dilutions) and reactive in specific Treponema Pallidum particle agglutination and Treponema enzyme-linked immunosobent assays (IgG positive, IgM negative). The CSF was acellular with normal protein and glucose, and tests for syphilis were negative for non-treponeal(TRUST) and reactive in specific Treponema Pallidum particle agglutination and Treponema enzyme-linked immunosobent assays(IgG positive, IgM negative). The oligoclonal IgG bands(OB) and anti-NMDAR was positive in CSF and serum. Cranial MRI showed lateral ventricles and the third ventricle enlargement (Fig. 4) and focal high T2/FLAIR signal abnormality involving bilateral temporal lobe, corona radiate and centrum semiovale (Figs. 5 and 6). We thought the patient may present 2 diseases: neurosyphilis and anti-NMDAR encephalitis. Besides penicillin G, pulsed methylprednisolone plus intravenous immunoglobulin was administered. He was stable afterwards.

## Conclusions

The NMDAR contributes to excitatory synaptic transmission across many brain regions. It plays a key role in neurodevelopment and synaptic plasticity. A highly active NMDAR is composed of glycine/D-serine-binding NR1 subunit and multiple glutamate-binding NR2 subunits. The hyperactivation of NMDAR has been shown to induce acute neuronal death and chronic neurodegeneration, while the hypoactivation of NMDAR is related to the development of psychiatric state [5].

The NR1 subunit is considered obligatory for functional NMDAR assemblies. Recent advances in genetic, preclinical and clinical pharmacological, and brain studies have shown the role of disrupted NMDAR-NR1 subunit-mediated glutamatergic pathways in schizophrenia [6]. *Psychosis* is a canonical symptom of anti-NMDAR encephalitis. In our case, Patient 1, 2 and 4 all manifested episodes of agitation.

Approximately three-quarters of CNS disorders with antibodies to surface antigens manifest in epileptic seizures [7]. Some epileptics who are not sensitive to conventional anticonvulsants may have an immune-mediated etiology [8, 9], and that epilepsy with psychiatric symptoms may have anti-NMDAR encephalitis [10]. Patient 1 presented with occasional seizures accompanying with psychosis.

Anti-NMDAR encephalitis is proved to be antibody-mediated [11, 12]. The NMDA receptor antibodies are IgGs directed against *extracellular* epitopes of the GluN1 subunit [13]. The immunopathological findings of anti-NMDAR encephalitis are increased deposits of immunoglobulin G and reactive microglial staining with anti-CD68 antibody, mainly in the basal forebrain, hippocampus, basal ganglion, and cervical spinal cord [2, 11, 13]. J.-P. Camdessanche thought perivascular inflammatory B-cell accumulation can appear in patients and play a positive role in brain T-cell infiltration, antibody secretion by plasmocytes, microglial and astro-glial proliferation [14].

Cui Li showed NMDAR played a critical role in regulation of oligodendrocyte precursor cells differentiation and remyelination [15]. Studies have indicated patients with anti-NMDAR encephalitis may develop episodes of demyelinating disorders, and conversely patients with NMO or demyelinating disorders in atypical types may have anti-NMDAR encephalitis [16, 17].

*Patient 1 had 6 relapses in 28 years. He presented with seizures and dysarthria and movement disorder. We think the seizure of his 9-year-old is the first event. The delay range between relapses is 3–16 years. The level of anti-NMDAR antibodies in CSF and serum decreased when patient 1 show substantial clinical recovery. There are still different opinions on relationship between the level of anti-NMDAR and disease activity. Some researches indicate this relationship exists* [13, 18, 19]*, while some show they are uncorrelated* [20, 21]*.*

Patient 2 had a relapsing disease course of CNS(central nervous system) demyelinating disorders. She presented with three subacute episodes of spinal cord and brainstem symptoms, which responded to steroid treatment. In view of MRI, cord spinal T2 lesions non-suggestive of MS, and tests for anti-aquaporin 4(AQP4) antibodies in serum and CSF were weakly positive, we diagnosed her neuromyelitis optica spectrum disorder(NMOSD). Although brainstem syndromes and short myelitis lesions [22] were reported in NMOSD [23, 24], we looked for other possible disorders involving auto-immune encephalitis and found anti-NMDAR-Ab in CSF and serum before steroid was applied.

Patient 1 and patient 2 both showed a relapsing disease course. In anti-NMDAR encephalitis, Relapse rate is reported to be 20-30%. [13, 25, 26]. At relapses, typical syndromes were usually lacking [27]. It can be separated by intervals of months or years. Between relapses is substantial recovery. Relapse rates may be higher in patients without immunotherapy during the first episode [18, 27] and in patients without detectable tumors [4, 18], suggesting importance of early immunotherapy. Differentiating from disorders with antibodies to intracellular antigens(Hu,Ri,Yo,Ma_2_ and amphyphism antibodies), which is due to T-cell mediated cytotoxity, poorly responsive to immunotherapy, has a progressive course and its treatment is directed to the underlying malignancy [28], disorders with antibodies to cell surface antigens(VGKC-complex,NMDAR) may work by antibody-binding, internalization, and loss of the target antigen [29], are often sensitive to treatment [4, 30], have a relapsing course, have a better prognosis, and are less commonly paraneoplastic [28, 31].

Patient 3 and Patient 4 were confirmed neurosyphilis, with anti-NMDAR antibody positive in CSF and serum. We have not found any reports about neurosyhilis accompanying with anti-NMDAR antibody so far.

We speculate the vision decrease of patient 3 may be caused by neurosyphilis. Patient 4 was diagnosed paralytic dementia. His MRI showed the lateral and third ventricles enlargement, which is associated with schizophrenia [32]. The oligoclonal IgG bands(OB) in CSF while not demonstrable in corresponding serum reflected a local B-cell response following central nervous system(CNS) inflammation. We cannot exclude that neurosyphilis has led to secondary immunological response of anti-NMDAR-Ab production. It’s also possible that the patient may have presented 2 diseases concurrently: neurosyphilis and anti-NMDAR encephalitis. Such patients provide a compelling argument to pursue the possible relation of neurosyphilis and anti-NMDAR antibody.

This study has several limitations. The follow-up is relatively short. We cannot know if there are relapses of anti-NMDAR encephalitis in neurosyphilis. We recently gave patient 1 mycophenolate mofetil after 6 relapses, so we cannot know if that will prevent relapse. Since patient 1 had seizures every 3–5 years, it’s a question whether anti-epileptic drugs are given initially.

## Abbreviations

AQP4: Anti-aquaporin 4
CEA: Carcinoembryonic antigen
CSF: Cerebrospinal fluid
EEG: Electroencephalography
ENA: Extractable nuclear antigen antibody
FLAIR: Flow attenuated inversion recovery
MBP: Myelin basic protein
MRI: Magnetic resonance imaging
MS: Multiple sclerosis
NMDAR: N-methyl-D-aspartate receptor
NMO: Neuromyelitis optica
NMOSD: Neuromyelitis optica spectrum disorder
OB: Oligoclonal bands
PCR: Polymerase chain reaction
TRUST: Syphilis toluidine red untreated serum test
VGKC: Voltage-gated potassium channel-complex
